# Different assessment tools to detect sarcopenia in patients with Parkinson's disease

**DOI:** 10.3389/fneur.2022.1014102

**Published:** 2022-11-28

**Authors:** Dora Valent, Marina Peball, Florian Krismer, Anna Lanbach, Sophie Zemann, Corinne Horlings, Werner Poewe, Klaus Seppi

**Affiliations:** Department of Neurology, Medical University of Innsbruck, Innsbruck, Austria

**Keywords:** Parkinson's disease, sarcopenia, prevalence, screening tool, sensitivity, specificity

## Abstract

**Introduction:**

Sarcopenia and Parkinson's disease are closely related diseases of the elderly population leading to progressive disability and nursing-dependent care.

**Objective:**

The aim of this study was to estimate the prevalence of sarcopenia in PD patients with three different approaches: (1) the screening tool SARC-F, (2) EWGSOP-1 criteria, and (3) EWGSOP-2 criteria. Moreover, we aimed to evaluate the diagnostic accuracy of the screening tool SARC-F to detect sarcopenia according to the updated EWGSOP-2 criteria.

**Methods:**

Eighty-one patients with Parkinson's disease aged 65 years and above were interviewed in a cross-sectional study at a tertiary referral center. All patients were screened with the SARC-F questionnaire and were evaluated for motor and non-motor symptoms, exercise, quality of life, and frailty. Muscle mass was assessed with bioelectrical impedance analysis, handgrip strength with a dynamometer, and gait speed was assessed with the 8-m walk test. EWGSOP-2 criteria were considered the gold standard to diagnose sarcopenia in our study.

**Results:**

Eighty-one patients were evaluated (mean age: 73.82; SD 5.30). The prevalence of sarcopenia was 28.4% according to the EWGSOP-2 criteria. The concordance between EWGSOP-2 and EWGSOP-1 was poor (weighted kappa of 0.361[95% 0.164–0.557]). The sensitivity of the SARC-F screening test for detecting sarcopenia was 60.9%. The corresponding AUC in the ROC curve analysis showed 0.598 (0.462, 0.734 CI). The item assessing strength was found to have the highest sensitivity (69.6%).

**Conclusion:**

Sarcopenia prevalence in patients with PD in Tirol, Austria is higher with EWGSOP-1 criteria compared to EWGSOP-2 criteria. The sensitivity and specificity of the SARC-F scale to detect sarcopenia in this population are poor.

## Introduction

Sarcopenia is a skeletal muscle disorder manifesting in the progressive loss of muscle mass and strength leading to adverse health outcomes including, fractures and falls, lower quality of life, and mortality (1). Until 2019, pre-sarcopenia was operationalized by the European Working Group for Sarcopenia in Older People (EWGSOP) as decreased muscle mass. Sarcopenia was confirmed upon the addition of either decreased muscle strength or gait speed and a severe sarcopenic stage was fulfilled when all three components were met (i.e., EWGSOP-1 criteria) (2). In 2019, EWGSOP published a new set of criteria (i.e., EWGSOP-2), whereby low muscle strength, instead of muscle mass, was named the new primary indicator of sarcopenia, with low muscle mass confirming the diagnosis. Decreased gait speed renders sarcopenia severe. A systematic review estimated the prevalence to be 1–29% in the general population according to the EWGSOP-1 criteria (3). With EWGSOP-2 criteria, the prevalence decreased, with some studies reporting occurrences ranging from 0.2 to 13.7% (4–9). Agreement between the two criteria has been mostly inconsistent with some studies showing good (5, 10, 11) and most showing poor agreement (6, 8, 12–15). It was also suggested by EWGSOP to use the validated screening scale SARC-F to identify potential patients who might be suffering from sarcopenia (1). Using the SARC-F scale, 6–65% of the general elderly were found to be at risk for sarcopenia (16–23). Despite its ease of use in clinical settings, the validity of the SARC-F has been debated due to its low sensitivity (17, 24).

Sarcopenia and Parkinson's disease (PD) are both age-related disorders that may share a common underlying pathway (25), in at least a subpopulation of both diseases, leading to progressive disability through motor and non-motor symptoms. Elevated neuro-inflammatory mediators (26), reduction in grey matter in specific brain regions (27), reduced motor neuron units in the hand (28, 29), and altered testosterone levels (30) are examples of markers associated with both sarcopenia and PD. Moreover, sarcopenia has been reported to be more prevalent in this patient population which makes this an imminent topic for clinical prevention and research (31). Currently, no study has assessed the prevalence of sarcopenia in PD using the most recent EWGSOP criteria (EWGSOP-2) with the suggested European cut-off points, and the research conducted so far has yielded conflicting results.

The aim of this study was to estimate the proportion of PD patients with sarcopenia according to three different assessment tools, namely the SARC-F questionnaire, EWGSOP-1 criteria, and EWGSOP-2 criteria. Moreover, we sought to determine the sensitivity and specificity of the SARC-F questionnaire in this patient demographic to detect sarcopenia according to the most recent EWGSOP-2 criteria.

## Materials and methods

### Study population

This was an observational, cross-sectional study in which patients with PD aged 65 years or older were recruited consecutively from the movement disorders outpatient clinic and neurological wards of the Medical University of Innsbruck between 2018 and 2021. In order to be included, all patients had to be diagnosed with PD according to the Movement Disorders Society clinical diagnostic criteria (32) and sign an informed consent form. The presence of another movement disorder or any contraindications to the bioelectrical impedance analysis (BIA) machine, such as deep brain stimulation and an apomorphine pump, were exclusion criteria.

### Sarcopenia assessment

Sarcopenia was assessed by three different methods: SARC-F, EWGSOP-1, and EWGSOP-2 criteria. Patients were administered the SARC-F, a simple screening questionnaire composed of five items: strength, climbing stairs, assistance with walking, rising from a chair, and falls (33). Each item is scored on a scale from 0 to 2 with a total score of 0–10 points. Because a score of 4 or greater is predictive of sarcopenia, patients with a score of 4 or greater were classified as sarcopenic, while those with a score of <4 were classified as non-sarcopenic. Diagnosis of sarcopenia according to the EWGSOP-1 criteria was the following: low muscle mass to be considered pre-sarcopenic, detection of low muscle strength or low gait speed to confirm sarcopenia, and fulfillment of all three criteria to be considered severely sarcopenic (2). Diagnosis of sarcopenia established by EWGSOP-2 criteria was the following: sarcopenia was probable when low muscle strength was detected, the addition of low muscle mass confirmed sarcopenia and the addition of low gait speed made the diagnosis severe (1). Respective cut-off values are shown in Table 1.

**Table 1:** EWGSOP-1 and EWGSOP-2 cut-off points for sarcopenia.

**Criterion**	**EWGSOP-1 cut-off points**	**EWGSOP-2 cut-off points**
Handgrip strength	Men: <30 kg	Men: <27 kg
	Women:<20 kg	Women: <16 kg
Muscle mass BIA	SM/height^2^	ASM/height^2^
	Men: 8.87 kg/m^2^	Men: <7.0 kg/m^2^
	Women: 6.42 kg/m^2^	Women: <5.5 kg/m^2^
Gait speed	<0.8 m/s	≤0.8 m/s

EWGSOP, European working group for sarcopenia in older people; BIA, bioelectrical impedance analysis; SM, skeletal muscle; ASM, appendicular skeletal muscle.

Muscle strength was assessed with a calibrated handheld dynamometer (CITEC CT3002). Two trials for each hand were performed and the best result from the strongest hand was used. Muscle mass was measured with the InBody 770 BIA machine at 50 kHz. Appendicular muscle mass was calculated based on the recommended equation developed by Sergi et al. (34). Walking speed, measured in meters/second (m/s), was evaluated by the 8-m gait test, where the participant walked along a straight 8-meter track and the time was measured by a stopwatch.

### Other variables

Demographic information (sex and age), anthropomorphic measurements (weight and height), disease duration, comorbidities (Charlson Comorbidity Index), and quality of life (PD Questionnaire-8) were collected. Disease stage and severity were established with the Hoehn and Yahr scale and the MDS-UPDRS.

### Statistical analysis

The data were analyzed with the aid of the SPSS program version 21 (IBM Corp., Armonk, NY, USA). Data are presented as mean plus or minus standard deviation (SD) for continuous and as a percentage for categorical variables. Prevalence rates are given in percentage with a 95% confidence interval (CI). Score values ranging from 1 to 10 points for the SARC-F test were reported as a dichotomized outcome of the assessment (≥4 as sarcopenic and <4 as non-sarcopenic). Continuous variables were tested for normality using the Kolmogorov–Smirnov test. Parametric data are expressed as mean and standard deviation and compared using Student's *t*-test. Non-parametric data were tested with the Shapiro–Wilk test and are expressed as mean and standard deviation. We used the area under the receiver operating characteristic (ROC) curve to test the predictive accuracy. Kappa statistic according to Fleiss–Cohen was calculated to ascertain the level of agreement between the classifications of sarcopenia according to the different screening tools.

## Results

### Demographic data

Demographic data of the cohort including sex differences are shown in Table 2. A total of 81 elderly patients with PD aged 73.8, SD 5.3 (44 men, 37 women) were included in the analyses. The mean duration of the disease was 11.5 ± 7.6 years and 39.5% were in H&Y stage III or above. No significant differences between gender were found regarding demographic and clinical characteristics (*p* > 0.05), except for in the number of comorbidities (*p* = 0.003) (Table 2).

**Table 2:** Sex differences in demographic and clinical characteristics.

	**Men**	**Women**	*p*-value^*^
	***N* = 44**	***N* = 37**	
	**Mean ±SD**		
Age (years)	72.6 ± 5.1	74.6 ± 5.4	0.692
Height (cm)	1.75 ± 0.0	1.63 ± 0.0	0.568
Weight (kg)	79.5 ± 13.6	66.9 ± 12.0	0.484
Disease duration (years)	12.0 ± 8.2	11.0 ± 6.8	0.406
CCI	0.5 ± 0.8	0.2 ± 0.6	0.003
LEDD (mg)	834.1 ± 461.1	695.3 ± 426.5	0.255
	***n*** **(%)**		***p*** -value^**^
H&Y I	3 (6.8)	2 (5.4)	
H&Y II	25 (56.8)	20 (54.1)	
H&Y III	10 (22.7)	9 (24.3)	
H&Y IV	6 (13.6)	6 (16.2)	
H&Y V	0 (0.0)	1 (2.7)	0.981
	**Median (IQR)**		***p*** -value^***^
MDS-UPDRS Part I	11 (7.0–15.0)	14 (11.0–18.0)	0.060
II	14 (8.25–24.5)	15 (8.0–22.5)	0.909
III	32.5 (23.3–42.8)	30 (23.0–44.0)	0.985
IV	1 (0.0–5.8)	3 (0.0–6.0)	0.826

Data are presented as mean plus or minus standard deviation for continuous variables or as median with interquartile range for quantitative categorical variables, or as stated.

CCI, Charlson Comorbidity Index; H&Y, Hoehn & Yahr; LEDD, Levodopa Equivalent Daily Dose; PDQ-8, Parkinson's Disease Questionnaire Summary Index; MDS-UPDRS, Movement Disorders Society- Unified Parkinson's Disease Rating Scale.

* Student's *t*-test.

** Fisher's exact test.

*** Mann–Whitney *U*-test.

### Prevalence of sarcopenia

Table 3 compares the prevalence of sarcopenia using SARC-F, EWGSOP-1, and EWGSOP-2 criteria. The prevalence of sarcopenia including severe sarcopenia according to SARC-F was 44.4%, with EWGSOP-1 criteria at 51.9% and with EWGSOP-2 criteria at 28.4%. Agreement between the two EWGSOP criteria was poor (Kappa = 0.361[95% CI 0.164–0.557], data not shown in tables).

**Table 3:** Proportion of probable sarcopenia and sarcopenia according to different assessment tools.

**Category sarcopenia**	**Total *n* = 81(%)**	**CI (95%)**	**Men *n* = 44(%)**	**CI (95%)**	**Women *n* = 37(%)**	**CI (95%)**
Probable (SARC-F)	32 (39.5%)	28.8–51.0	17 (38.6%)	24.4–54.5	15 (40.5%)	24.8–57.9
Probable (EWGSOP-1)	2 (2.5%)	0.3–8.6	2 (4.6%)	0.6–15.5	0 (0.0%)	0.0–9.5
Probable (EWGSOP-2)	44 (54.3%)	42.9–65.4	22 (50.0%)	34.6–65.4	22 (59.5%)	42.1–75.3
Sarcopenia^*^ (SARC-F)	36 (44.4%)	33.4–55.9	17 (38.6%)	24.4–55.9	19 (51.4%)	34.4–68.1
Sarcopenia^*^ (EWGSOP-1)	42 (51.9%)	40.5–63.1	28 (63.6%)	47.7–77.6	14 (37.8%)	22.5–55.2
Sarcopenia^*^ (EWGSOP-2)	23 (28.4%)	18.9–39.5	12 (27.3%),	15.0–42.8	11 (29.7%)	15.9–47.0

CI, confidence interval; SARC-F, a simple 5-item questionnaire for sarcopenia; EWGSOP-1, first version of the consensus of European Working Group on Sarcopenia in Older People (2010); EWGSOP-2, revised version of the consensus of European Working Group on Sarcopenia in Older People (2019).

* sarcopenia, including confirmed and severe.

### Sensitivity and specificity of SARC-F and its individual items

The sensitivity of the SARC-F screening test for detecting sarcopenia (as per EWGSOP-2 criteria) was 60.9% (Table 4). The corresponding AUC in the ROC Curve analysis showed 0.598 (0.462, 0.734 CI). The specificity of the test was poor (62.1%) (Table 4). All five items on the questionnaire had similar sensitivities individually (ranging from 56.5 to 69.6%, Table 5) compared to the test as a whole. The specificity of the individual items is all poor, with all values falling below 62.1%. The positive predictive values of all the individual items were under 40%, while the negative predictive values were all above 68%. Thus, a non-sarcopenic person has a probability of under 40% being categorized as at risk for sarcopenia by one of these items (Table 5).

**Table 4:** Sensitivity and specificity of SARC-F with predictive values according to EWGSOP-2.

***n***	**Sensitivity (%)**	**Specificity (%)**	**PV+**	**PV-**
81	60.9	62.1	0.4	0.8

PV+, positive predictive value; PV-, negative predictive value.

**Table 5:** Sensitivity of individual SARC-F items according to EWGSOP-2.

**SARC-F**	**True positive screening**	**Sarcopenia *n* = 23**		**PV+**	**PV-**
	***n* (%)**	**Sensitivity (%)**	**Specificity (%)**		
Strength	16 (19.8)	69.6	51.7	36.4	81.1
Assistance with walking	13 (16.0)	56.5	55.2	33.3	76.2
Standing up from chair	15 (18.5)	65.2	29.3	26.8	68.0
Climbing stairs	14 (17.2)	60.1	62.1	38.9	80.0
Falls	13 (17.3)	56.5	51.7	31.7	75.0

PV+, positive predictive value; PV-, negative predictive value.

## Discussion

Here, we show that the SARC-F screening questionnaire as a whole has poor sensitivity (60.9%) and specificity (62.1%) in the detection of sarcopenia in patients with Parkinson's disease in Tirol, Austria when followed up by an assessment with the most current EWGSOP-2 criteria. Studies assessing PD patients with the EWGSOP-2 criteria are scarce. In their Brazilian PD cohort, Campos Lima da Luz et al. found similar specificity (68.1%) but lower sensitivity (23.1%) (35). Our results show a balance between sensitivity and specificity that is rather indicative of a cut-off value of greater or equal to 2 on the SARC-F questionnaire (36). Although other studies report high specificity in populations without PD, in this patient population, SARC-F does not seem to correctly identify patients who do not have sarcopenia according to the EWGSOP-2 criteria, which means that necessary therapeutic implementations might be allocated to the wrong patients. Studies show that adding a calf measurement to the screening process improves the identification of truly patients with sarcopenia (22, 35, 37, 38). Assessing the individual items on the instrument yielded equally poor sensitivity results, with each item apart for strength, having poor to negligible sensitivity, comparable to previous findings in a PD cohort (35). The highest sensitivity was calculated for the item “strength” at 69.6%. The “falls” item in the questionnaire is especially confounding when it comes to evaluating sarcopenia in patients with PD as recurrent falls are already reported more often by patients with PD compared with age-matched controls (39) and are a sign of advancing PD. Tailoring the questions on the questionnaire to fit a PD patient profile, increasing the cut-off values, or adding a calf circumference measurement to increase accuracy may lead to higher sensitivity or specificity among patients with PD.

To our best knowledge, we are the first study to evaluate the prevalence of sarcopenia among a European population in patients with PD assessed according to the criteria and cut-offs suggested by EWGSOP in 2019. According to EWGSOP-2, 28.4% of participants had confirmed sarcopenia, with men and women having 27.3 and 29.7%, respectively. These results are in line with another study that assessed sarcopenia in Brazilian PD participants (35). The prevalence of sarcopenia based on EWGSOP-1 was 51.9% in total (63.6% in men and 37.8% in women). Other studies have assessed sarcopenia in a PD population according to EWGSOP-1 criteria report numbers between 6 and 55% and varying differences between sex ratios (29, 35, 40–43).

Applying the EWGSOP-2 criteria resulted in a lower prevalence of sarcopenia due to stricter recommended cut-off values for handgrip strength and muscle mass and the adaptation of the operational definition of sarcopenia. When comparing the two criteria, other studies on non-PD populations have also found that the EWGSOP-1 criteria lead to higher prevalence (6, 9, 15, 44). A recent review proposes that the difference in prevalence is about 7% (7). Whether this difference is an overestimation by EWGSOP-1 or an underestimation by EWGSOP-2 has to be proven in longitudinal follow-up studies assessing outcomes and disease milestones. However, the EWGSOP-2 criteria should be more accurate than the first version as it uses muscle strength as the primary diagnostic factor. Muscle strength has been shown to be a better predictor of the adverse health outcomes that describe sarcopenia, namely quality of life, disability, and mortality (1, 45). Specifically, it has been shown that out of the three criteria defining sarcopenia, muscle strength is a better predictor of future mobility issues than muscle mass (46) and that it is also associated with recurrent falls independent of muscle mass (47). Moreover, with EWGSOP-2, the number of confirmed and severe cases was lower in comparison to EWGSOP-1, but the prevalence of probable cases was significantly higher, suggesting that muscle strength is more sensitive in identifying probable cases of sarcopenia (44, 48).

A recently published meta-analysis suggests that although sarcopenia seems to be common in patients with PD, the current evidence does not allow for the conclusion of a definitive prevalence of sarcopenia in patients with PD (31). Sarcopenia, indeed, was also common in our Austrian PD population. This can be due to a plethora of associated factors and symptoms, such as gait hypokinesia, which is present from an early stage of the disease (49). Recurrent falls are also reportedly higher in PD and can lead to physical inactivity and disability (39, 50). A decrease in the number of motor neurons due to dopaminergic dysfunction may also contribute to a higher proportion of people with PD having sarcopenia (28, 41). Additionally, a loss of lean muscle mass due to malnutrition in advanced PD has also been linked to sarcopenia (51).

The proportion of sarcopenia based on the SARC-F questionnaire was 44.4% which falls within the reported 6–65% in the general population (16–24). Two other studies report SARC-F-based prevalence in PD of 55.8% (39) and 30% (35), respectively; however, the latter reports numbers with a cut-off value of greater or equal to 6, making comparison difficult.

This study has several limitations. First, our study was limited by small sample size and the fact that all patients were recruited from a single center. Second, our results might be confounded by a regional bias in Tirol, Austria, where the population is generally active into old age. Thus, it is important to note that the prevalence data reported here have a geographical limit. A future multicenter study could yield more replicable data. Moreover, our study lacks a control population to compare the prevalence of sarcopenia according to the EWGSOP-2 criteria between patients with PD and the general population. It seems, indeed, that sarcopenia was more common in our PD population compared to the general population described in previous articles, 1–29% EWGSOP-1 (3), 10–11% EWGSOP-2 criteria (7, 8). EWGSOP-2 also suggests developing regional cut-off values to account for stature-dependent muscle strength and gait speed, which have been implemented by several other groups for Brazilian and Iranian and Asian populations (6, 9, 15).

## Conclusion

We report the prevalence of sarcopenia in patients with PD in Tirol, Austria to be 28.4 % assessed with the EWGSOP-2 criteria. Our results are in line with other groups stating that EWGSOP-1 criteria overestimate sarcopenia prevalence compared to EWGSOP-2. Due to the low sensitivity and specificity of the SARC-F questionnaire, this scale is not conducive to detecting sarcopenia in a PD population.

## Data availability statement

The raw data supporting the conclusions of this article will be made available by the authors, without undue reservation.

## Ethics statement

The studies involving human participants were reviewed and approved by Ethikkommission der Medizinischen Universität Innsbruck. The patients/participants provided their written informed consent to participate in this study.

## Author contributions

DV: Research project: conception, organization, execution, Statistical analysis: design, execution, Manuscript: writing of the first draft. MP: Research project: conception, organization, execution, Statistical analysis: review and critique, Manuscript: review and critique. FK: Statistical analysis: execution, review and critique, Manuscript: review and critique. AL and SZ: Research project: execution. CH: Research project: conception, Manuscript: review and critique, WP: Research project: conception, KS: Research project: conception, Statistical analysis: review and critique, Manuscript: review and critique. All authors contributed to the article and approved the submitted version.

## Conflict of interest

The authors declare that the research was conducted in the absence of any commercial or financial relationships that could be construed as a potential conflict of interest.

## Publisher's note

All claims expressed in this article are solely those of the authors and do not necessarily represent those of their affiliated organizations, or those of the publisher, the editors and the reviewers. Any product that may be evaluated in this article, or claim that may be made by its manufacturer, is not guaranteed or endorsed by the publisher.
